# UDP-sugars activate P2Y_14_ receptors to mediate vasoconstriction of the porcine coronary artery

**DOI:** 10.1016/j.vph.2017.12.063

**Published:** 2018-04

**Authors:** Zainab S.B. Abbas, M. Liaque Latif, Natalia Dovlatova, Sue C. Fox, Stan Heptinstall, William R. Dunn, Vera Ralevic

**Affiliations:** a School of Life Sciences, University of Nottingham, Nottingham NG7 2UH, UK; b School of Medicine, University of Nottingham, Nottingham NG7 2UH, UK

**Keywords:** Coronary artery, P2Y_6_ receptor, P2Y_14_ receptor, UDP-glucose, UDP sugars, MRS2690, Sugar nucleotides, BSA, bovine serum albumin, DABCO, 1,4-diazabicyclo[2.2.2]octane, DAPI, 4′,6-diamidino-2-phenylindole, ERK, extracellular signal-regulated kinases, FITC, fluorescein isothiocyanate, MAPK, mitogen-activated protein kinases, PPTN, 4-((piperidin-4-yl)-phenyl)-7-(4-(trifluoromethyl)-phenyl)-2-naphthoic acid, RCF, relative centrifugal force, KCl, potassium chloride, MRS2578, *N*,*N*"-1,4-butanediyl*bis*[*N*'-(3-isothiocyanatophenyl)thiourea, MRS2690, diphosphoric acid 1-α-d-glucopyranosyl ester 2-[(4′-methylthio)uridin-5″-yl] ester disodium salt, NO, nitric oxide, L-NAME, Nω-nitro-L-arginine methyl ester, NTPDase1, nucleotide triphosphate diphosphohydrolase-1, OCT, optimal temperature cutting compound, PAGE, polyacrylamide gel electrophoresis, PBS, phosphate buffered saline, SDS, sodium dodecyl sulphate, SNP, sodium nitroprusside, TBST, Tris-buffered saline and Tween 20, VASP, vasodilator-stimulated phosphoprotein

## Abstract

**Aims:**

UDP-sugars can act as extracellular signalling molecules, but relatively little is known about their cardiovascular actions. The P2Y_14_ receptor is a G_i/o_-coupled receptor which is activated by UDP-glucose and related sugar nucleotides. In this study we sought to investigate whether P2Y_14_ receptors are functionally expressed in the porcine coronary artery using a selective P2Y_14_ receptor agonist, MRS2690, and a novel selective P2Y_14_ receptor antagonist, PPTN (4,7-disubstituted naphthoic acid derivative).

**Methods and results:**

Isometric tension recordings were used to evaluate the effects of UDP-sugars in porcine isolated coronary artery segments. The effects of the P2 receptor antagonists suramin and PPADS, the P2Y_14_ receptor antagonist PPTN, and the P2Y_6_ receptor antagonist MRS2578, were investigated. Measurement of vasodilator-stimulated phosphoprotein (VASP) phosphorylation using flow cytometry was used to assess changes in cAMP levels. UDP-glucose, UDP-glucuronic acid UDP-*N*-acetylglucosamine (P2Y_14_ receptor agonists), elicited concentration-dependent contractions of the porcine coronary artery. MRS2690 was a more potent vasoconstrictor than the UDP-sugars. Concentration dependent contractile responses to MRS2690 and UDP-sugars were enhanced in the presence of forskolin (activator of cAMP), where the level of basal tone was maintained by addition of U46619, a thromboxane A_2_ mimetic. Contractile responses to MRS2690 were blocked by PPTN, but not by MRS2578. Contractile responses to UDP-glucose were also attenuated by PPTN and suramin, but not by MRS2578. Forskolin-induced VASP-phosphorylation was reduced in porcine coronary arteries exposed to UDP-glucose and MRS2690, consistent with P2Y_14_ receptor coupling to G_i/o_ proteins and inhibition of adenylyl cyclase activity.

**Conclusions:**

Our data support a role of UDP-sugars as extracellular signalling molecules and show for the first time that they mediate contraction of porcine coronary arteries via P2Y_14_ receptors.

## 1 Introduction

Cellular release of purine nucleotides, including UTP and ATP, is promoted by stimuli including hypoxia and inflammation leading to activation of cell-surface P2 receptors [8]. UDP-glucose can also be released from cells to act as an extracellular signalling molecule [32], [35], [36]. In preliminary studies we showed that UDP-glucose causes constriction of the porcine coronary artery [1], [2], [3]. Excessive contraction of the coronary circulation following hypoxic or inflammatory release of UDP-glucose would exacerbate the detrimental effects of these insults. This led us to investigate the hypothesis that the P2Y_14_ receptor, a receptor for UDP-glucose and UDP, is expressed in, and mediates the vasomotor role of UDP-sugars in the porcine coronary vasculature. Identification of the receptor(s) involved in contractile responses to UDP-sugars may have therapeutic potential in ischaemic heart disease.

P2Y receptors are G protein-coupled receptors activated by purine, pyrimidine and sugar nucleotides. There are eight members of the P2Y family, namely P2Y_1_, P2Y_2,_ P2Y_4,_ P2Y_6,_ P2Y_11,_ P2Y_12,_ P2Y_13_ and P2Y_14_ receptors, with the P2Y_14_ receptor being the most recently described [4], [9]. The P2Y_14_ receptor is a G_i/o_ protein-coupled receptor activated by UDP-sugars including UDP-glucose, UDP-galactose, UDP-glucuronic acid, and UDP-*N*-acetylglucosamine [4], [5], [16], [17], [23], [44], [45], [46]. The P2Y_14_ receptor is also activated by UDP, and by MRS2690, a more selective agonist that has a 7-fold greater potency at the P2Y_14_ receptor than UDP-glucose [31]. Quantitative analysis has revealed that the P2Y_14_ protein is expressed in many regions including the brain, adipose tissue, lung, spleen, heart and certain immune and inflammatory cells [9].

There is evidence for the expression of P2Y_14_ receptor transcript and protein in vascular smooth muscle cells [5], [19], [38], but relatively little is known about the functional expression of this receptor in the cardiovascular system. Until recently, the lack of availability of P2Y_14_ receptor antagonists made it difficult to determine a role for the P2Y_14_ receptor. A novel non-nucleotide compound_,_ 4-((piperidin-4-yl)-phenyl)-7-(4-(trifluoromethyl)-phenyl)-2-naphthoic acid (PPTN), was reported as a high-affinity competitive antagonist at the P2Y_14_ receptor [43]. PPTN inhibited UDP-glucose and MRS2690 mediated mast cell degranulation, and UDP-glucose mediated chemotaxis of neutrophils [6], [16], [17]. We recently used PPTN to show, for the first time, that UDP-glucose mediates vasoconstriction through activation of P2Y_14_ receptors in porcine pancreatic arteries [5]. UDP-glucose mediates contraction in mouse coronary arteries, and the response was retained in P2Y_2_ knockouts, however since neither a P2Y_14_ selective agonist nor antagonist were employed the identity of the receptor involved requires confirmation [21]. The aims of the current study were to determine whether UDP-sugars can modulate contractility of the porcine coronary artery and to characterize the P2 receptor(s) involved.

Since the P2Y_14_ receptor is G_i/o_ protein-coupled, this makes its investigation difficult in isolated tissues where cAMP levels are normally low. Accordingly, we used forskolin to stimulate adenylyl cyclase and raise cAMP levels, a method described by us [5] and others [42] previously to investigate vascular G_i/o_ protein-coupled receptors, and on this background we were able to investigate the pharmacology of the responses to UDP-glucose and other P2Y_14_ receptor agonists in the porcine coronary artery. In this study we report the functional expression of a contractile P2Y_14_ receptor sensitive to UDP-sugars in the porcine coronary artery. The contractile response was mimicked by the selective P2Y_14_ agonist, MRS2690, and the response was blocked by the selective P2Y_14_ receptor antagonist PPTN. Measurement of VASP phosphorylation, and of contractile responses in the presence of forskolin, indicated that UDP-glucose inhibits cAMP levels, consistent with the known G_i/o_ protein-coupling of the P2Y_14_ receptor.

## 2 Methods

### 2.1 Tissue preparation

Hearts were obtained from pigs (either sex, ~ 6 months old, weight ~ 50 kg) and were transported on ice from a local abattoir. The size and arterial system of the porcine heart are very similar to that of humans [49]. Porcine left circumflex coronary arteries were dissected out and stored overnight at 4 °C in oxygenated (95% O_2,_ 5% CO_2_) Krebs-Henseleit buffer (mM): sodium chloride 118; potassium chloride 4.8; magnesium sulphate 1.1; sodium hydrogen carbonate 25; potassium hydrogen phosphate 1.2; glucose 12; calcium chloride 1.25. A fine dissection was carried out the following day. Four adjacent 5 mm ring segments were cut from each vessel and mounted onto wires for isometric tension recording (Grass FT03 transducers) in warmed (37 °C) and gassed (95% O_2,_ 5% CO_2_) Krebs-Henseleit buffer in organ baths. In some arteries the endothelium was removed by gentle rolling of the segment with fine-tipped forceps inserted into the lumen [41]. The success of the treatment was evaluated by testing responsiveness to an endothelium-dependent vasodilator, substance P.

### 2.2 Responses in the porcine coronary artery

Rings were put under an initial tension of 10 g [41] and left for ~ 45 min to equilibrate, after which viability was assessed by eliciting contractions with 60 mM KCl. After washout and equilibration (30 min) cumulative concentration response curves to UDP-glucose (0.1 μM-1 mM), MRS2690 (1 nM-10 μM), UDP-glucuronic acid (0.1 μM-1 mM), and UDP-*N*-acetylglucosamine (0.1 μM-1 mM) were constructed at basal tone.

### 2.3 Forskolin elevation of cAMP

In some experiments, arteries were preconstricted with U46619 (5–10 nM), a thromboxane A_2_ mimetic, to elicit contraction of 35–75% of that to KCl. Forskolin (0.1 μM; activates adenylyl cyclase) was used to reverse the U46619-mediated contraction. The combination of forskolin and U46619 has previously been used to uncover α_2_-adrenoceptor-mediated contractions in porcine ear artery [42]. When vessels had reached a stable tone (30–45 min), cumulative-concentration response curves to UDP-glucose, MRS2690, UDP-glucuronic acid and UDP-*N*-acetylglucosamine were constructed. In separate experiments, PPTN (1 μM) was added 10 min prior to addition of U46619. Cumulative concentration-response curves to UDP-glucose, MRS2690 and UTP were constructed in the absence and presence of PPTN (1 μM). The effects of MRS2578 (10 μM, a P2Y_6_ receptor antagonist), and the P2 receptor antagonists PPADS (10 μM) and suramin (100 μM), were also investigated. In some experiments sodium nitroprusside (0.1 μM; activates guanylyl cyclase) was used instead of forskolin, together with U46619, to investigate effects on contractility to UDP-glucose. In some experiments UDP-glucose was treated with apyrase (5 U/ml, 30 min) before use and apyrase (5 U/ml) was also added to the organ baths prior to U46619 and forskolin additions.

### 2.4 VASP phosphorylation assay

Porcine coronary arteries were set up in organ baths as described above. After addition of U46619 (5–10 nM) and forskolin (0.1 μM), vessels were left to relax to basal tone. These were control vessels, which were paired with vessels where either 100 μM or 1 mM UDP-glucose, or 10 μM MRS2690, were added in the absence and presence of PPTN (1 μM). After the contractions had reached a maximum, vessels were removed, put into lysis buffer, and frozen in dry ice. Tissues were homogenized and the supernatant was separated by centrifugation (3000 RCF, 10 min). VASP-phosphorylation in the tissues was measured by flow cytometry using a cytometric bead assay where VASP in the samples was captured onto beads using monoclonal anti-VASP (IE273) followed by detection using FITC conjugated anti-VASP (5C6 anti-VASP pSer 157, Acris Antibodies, Germany) [27].

### 2.5 Preparation of platelets

Venous blood was obtained from 7 healthy volunteers (from School of Life Sciences, University of Nottingham). Ethics was approved by Thrombosis and Haemostasis Research Group, University of Nottingham. Blood samples from healthy volunteers were taken in accordance with local ethical permission and the declaration of Helsinki. Blood was immediately put into collecting tubes containing EDTA (4 mM) to prevent blood clotting. For platelet preparation, platelet rich plasma was obtained by centrifugation at 1100 RPM (15 min) followed by a 2000 RPM (20 min) centrifugation step to obtain a platelet pellet. Platelets were resuspended in sterile saline solution (0.9% sodium chloride) before centrifuging at 1600 RMP (15 min). The pellet was resuspended in 2 ml of saline. Cells were counted on Sysmex KX-21 Haematology analyser (Kobe, Japan). Cells were between 78.3 and 92.9% pure (contamination with red and white blood cells).

### 2.6 Platelet addition to porcine coronary arteries

Porcine coronary arteries were prepared as previously described. In some vessels the P2Y_14_ receptor antagonist PPTN (1 μM) was added 10 min prior to U46619 addition. After a stable tone had been established, isolated platelets were added to the vessels; the final platelet concentration in the bath was approximately 24,000–30,000/μL. In other experiments, L-NAME (100 μM) was added to porcine coronary arteries and left for 10 min before adding PPTN (1 μM). After precontraction with U46619, the isolated platelets were added.

### 2.7 Materials

Acetylcholine, apyrase (Grade III), histamine, uridine 5′-diphosphoglucose disodium salt (UDP-glucose), uridine 5′-diphosphoglucuronic acid trisodium salt (UDP-glucuronic acid), uridine 5′-diphospho-*N*-acetylglucosamine sodium salt (UDP-*N*-acetylglucosamine), uridine 5′-triphosphate trisodium salt hydrate (UTP), 7-beta-acetoxy-8,13-epoxy-1-alpha, 6-beta, 9-alpha-trihydroxylabd-14-en-11-one; coleonol (adenylyl cyclase activator: forskolin), and Nω-nitro-L-arginine methyl ester hydrochloride (L-NAME) were all obtained from Sigma Aldrich. Diphosphoric acid 1-α-d-glucopyranosyl ester 2-[(4′-methylthio)uridin-5″-yl] ester disodium salt (MRS2690) and *N*,*N*″-1,4-Butanediyl*bis*[*N*′-(3-isothiocyanatophenyl)thiourea] (MRS2578) were obtained from Tocris Biosciences Ltd. PPTN was a gift from Merck Frosst Centre for Therapeutic Research.

### 2.8 Statistical analysis

Data were fitted using non-linear regression and statistical analyses were performed using GraphPad Prism 6.0 software. R_max_ is the maximal response and logEC_50_ is the log of the molar concentration of agonist required to generate 50% of the R_max_. Differences between R_max_ and logEC_50_ values were compared using the extra sum of squares F test, by comparing the extra sum of squares that resulted from the analysis with separate R_max_ or logEC_50_ values; where out-of-sample extrapolation occurred all parameters were selected to determine if one curve adequately fit all data sets (Prism 6). Individual R_max_ and logEC_50_ values were also determined after curve fitting of data for each artery segment. These parameters were compared using one- or two-way ANOVA with Tukey's multiple comparisons post hoc test to determine which groups the differences were between when > 2 groups were being compared. Data from VASP-phosphorylation experiments were analysed using one-way ANOVA with Tukey's post test or Student's *t*-test. Data from platelet experiments were analysed by two-way ANOVA with repeated measures, with Fisher's least significant difference post hoc test. *P* < 0.05 was considered statistically significant. All data are presented as mean ± S.E.M. n signifies the number of animals.

## 3 Results

### 3.1 P2Y_14_ receptor agonists constrict coronary arteries

**Fig. 1:**
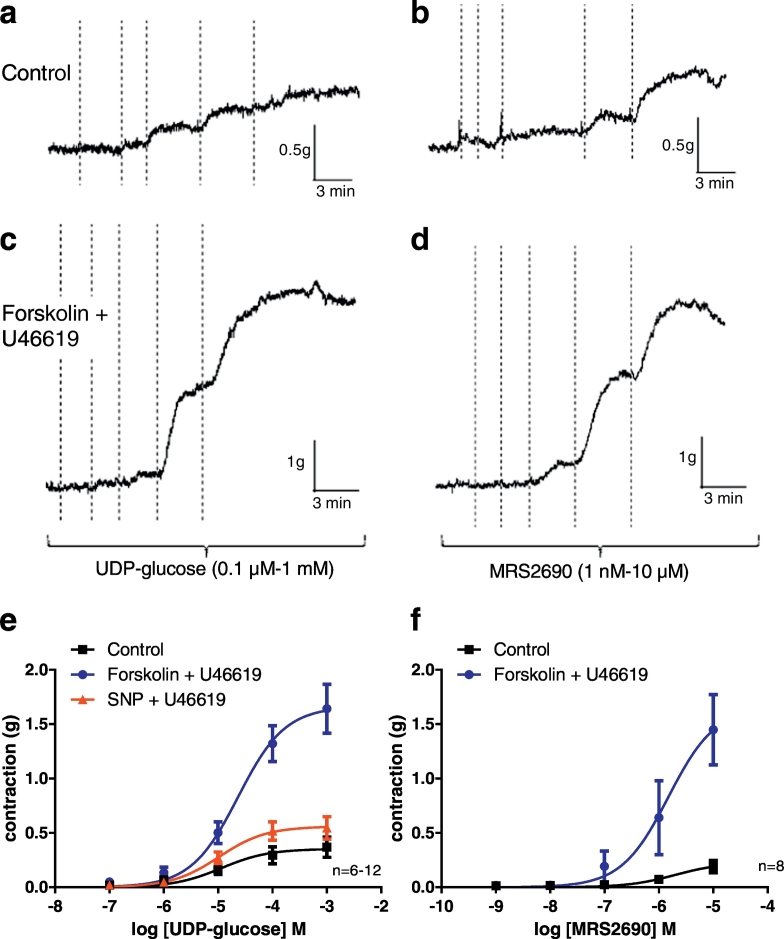
UDP-glucose and the selective P2Y_14_ receptor agonist MRS2690 mediate contraction of porcine coronary arteries. Representative traces showing concentration-dependent contractile responses of porcine coronary arteries to UDP-glucose (a, c) and MRS2690 (b, d) at basal tone in the absence (Control) and presence of forskolin (0.1 μM) plus U46619. Lines on traces represent addition of cumulative concentrations of agonists. The mean data ± S.E.M. are of experiments from 6 to 12 different pigs (e, f). For UDP-glucose (e) the R_max_ was significantly different between the groups (*P* < 0.001, extra sum of squares F test) with no difference in logEC_50_values. For MRS2690 (f) the curves were significantly different (P < 0.001, extra sum of squares F test).

The P2Y_14_ receptor is activated by various sugar nucleotides, including UDP-glucose, UDP-glucuronic acid and UDP-*N*-acetylglucosamine [4] and, more potently, by the synthetic analogue MRS2690 [31]. To identify and characterize the P2Y_14_ receptor in the porcine coronary artery, cumulative concentration-response curves to UDP-glucose (0.1 μM-1 mM) and MRS2690 (1 nM-10 μM) were initially constructed at basal tone. The agonists were found to produce concentration-dependent contractions (Fig. 1). MRS2690 was found to be more potent than UDP-glucose; R_max_ and EC_50_ values are reported in Table 1. Since the P2Y_14_ receptor is a G_i/o_ protein-coupled receptor mediating inhibition of adenylyl cyclase activity and hence reducing cAMP production [44], [45], [46], arteries were pre-exposed to forskolin to enhance intracellular cAMP levels prior to constructing the concentration-response curves. U46619 (5–10 nM), a thromboxane A_2_ mimetic, was additionally present in these experiments to maintain a stable basal tone (since forskolin induces vasorelaxation). Under these conditions, contractile responses to UDP-glucose and MRS2690 were enhanced as shown in Fig. 1. When the experiment was carried out using U46619 together with sodium nitroprusside (SNP, activator of guanylyl cyclase) instead of forskolin, no augmentation of the responses to UDP-glucose was observed, consistent with a specific involvement of cAMP (Fig. 1e). There was a significant difference in the R_max_ values for UDP-glucose between the groups (*P* < 0.001; extra sum of squares F-test). Analysis of the data (ANOVA with Tukey's post hoc test) showed that there was a significant increase in the R_max_ in the presence of forskolin plus U46619 (R_max_ 1.69 ± 0.24 g, *n* = 6) compared to the controls (R_max_ 0.35 ± 0.09 g, n = 6; P < 0.001) and compared to the presence of SNP plus U46619 (R_max_ 0.57 ± 0.1 g, *n* = 12; P < 0.001). The logEC_50_ values for UDG-glucose were similar under the different experimental conditions: control − 5.4 ± 0.41 (n = 6), forskolin plus U46619 − 4.66 ± 0.14 (n = 6) and SNP plus U466199 − 5.03 ± 0.08 (n = 12).

**Table 1:** Contractile responses of the porcine coronary artery to UDP-sugars. Shown are the EC_50_ (− log M) and R_max_ values.

Agonist	EC_50_ ± S.E.M (− log M)		R_max_ ± S.E.M (contraction in g)	
	Control	Forskolin (+ U46619)	Control	Forskolin (+ U46619)
UDP-glucose	4.89 ± 0.21 n = 12	4.66 ± 0.14 n = 6	0.37 ± 0.06	1.69 ± 0.24^#^
UDP-glucuronic acid	4.80 ± 0.27 *n* = 7	3.63 ± 0.21 n = 7	0.42 ± 0.04	2.26 ± 0.51^###^
UDP-*N*-acetylglucosamine	4.86 ± 0.23 n = 7	4.27 ± 0.11 n = 7	0.49 ± 0.10	1.72 ± 0.31
MRS2690	6.30 ± 0.45** n = 4	5.71 ± 0.16^ϕ^ *n* = 12	0.48 ± 0.14	1.77 ± 0.32

Contractile response curves were investigated in arteries under control conditions (Control) and in arteries to which forskolin had been added to stimulate adenylyl cyclase (with U46619 added to maintain a stable tone; see Methods) (forskolin). **P < 0.01 for MRS2690 −logEC_50_ compared with -logEC_50_s of UDP-glucose, UDP-glucuronic acid and UDP-*N*-acetylglucosamine in controls (two way ANOVA with Tukey's post test). ϕ P < 0.05, P < 0.001, P < 0.001 for MRS2690 -logEC_50_ compared with -logEC_50_s of UDP-glucose, UDP-glucuronic acid and UDP-*N*-acetylglucosamine respectively, in the presence of forskolin (two-way ANOVA with Tukey's post test). Agonist R_max_ values in the presence of forskolin were significantly greater compared to corresponding R_max_ values obtained under control conditions for both UDP-glucose and UDP-glucuronic acid; ^#^*P* < 0.5, ^###^P < 0.001 (two-way ANOVA with Tukey's post test). Data are mean ± S.E.M.

**Fig. 2:**
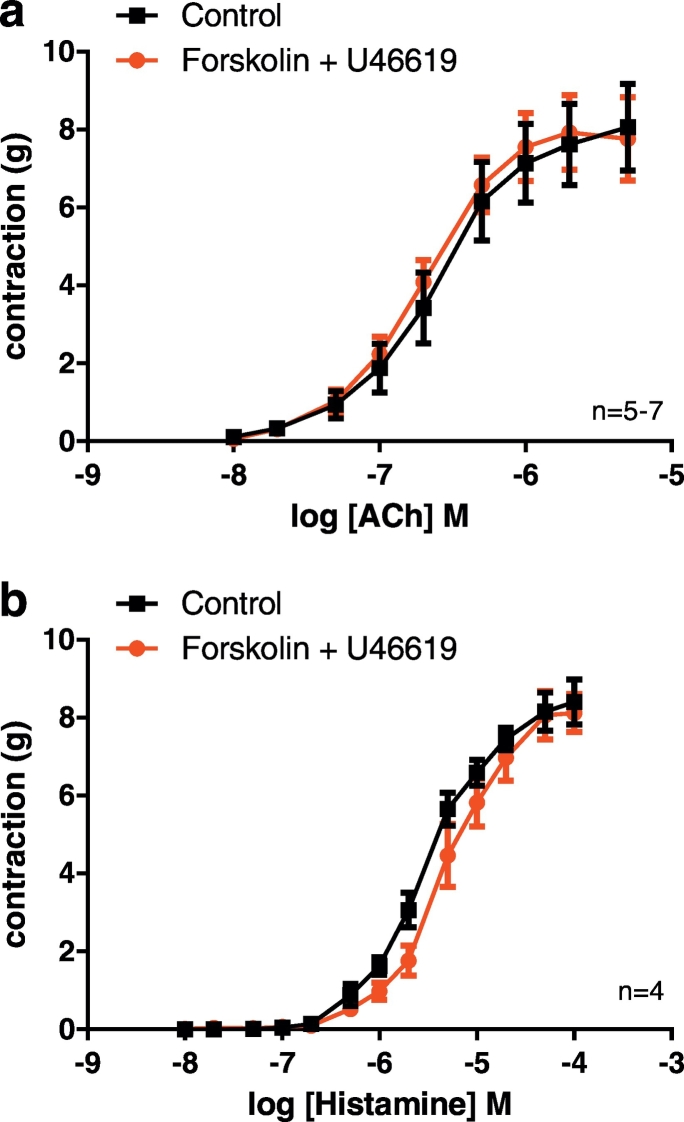
Forskolin plus U46619 do not augment contractions to acetylcholine and histamine in porcine coronary arteries. Cumulative concentration-response curves to acetylcholine (ACh) (a) and histamine (b), under control conditions and the effect of forskolin (0.1 μM) plus U46619. There was no significant difference in the R_max_ values for either compound, or in the logEC_50_ values for ACh, but there was a small but significant difference in logEC_50_ values for histamine, indicating a slightly reduced potency in the presence of forskolin plus U46619 (*P* < 0.01, extra sum of squares F-test). Data are mean ± S.E.M.

The combination of forskolin plus U46619 failed to augment contractile responses to histamine and acetylcholine (Fig. 2), which cause vasoconstriction of porcine coronary arteries primarily via G_q/11_ coupled H_1_ histamine receptors [22] and M_3_ muscarinic receptors [13]. R_max_ values for ACh were similar in the absence and presence of forskolin plus U46619: 8.94 ± 1.07 g (*n* = 5) and 9.53 ± 1.13 g (*n* = 6) respectively. logEC_50_ values were also similar in the absence and presence of forskolin plus U46619: −6.50 ± 0.08 (n = 5) and −6.57 ± 0.1 (n = 6), respectively (Fig. 2a). R_max_ values for histamine were similar in the absence and presence of forskolin plus U46619: 8.84 ± 0.52 g (*n* = 4) and 9.71 ± 0.8 g (n = 4), respectively. There was, however, a small but significant difference in the logEC_50_ values for histamine in the presence of forskolin plus U46619 compared to control conditions: −5.15 ± 0.1 (n = 4) and −5.45 ± 0.07 (n = 4), respectively (*P* < 0.01, extra sum of squares F-test) (Fig. 2b), indicating a small reduction in the potency of histamine in the presence of forskolin plus U46619.

**Fig. 3:**
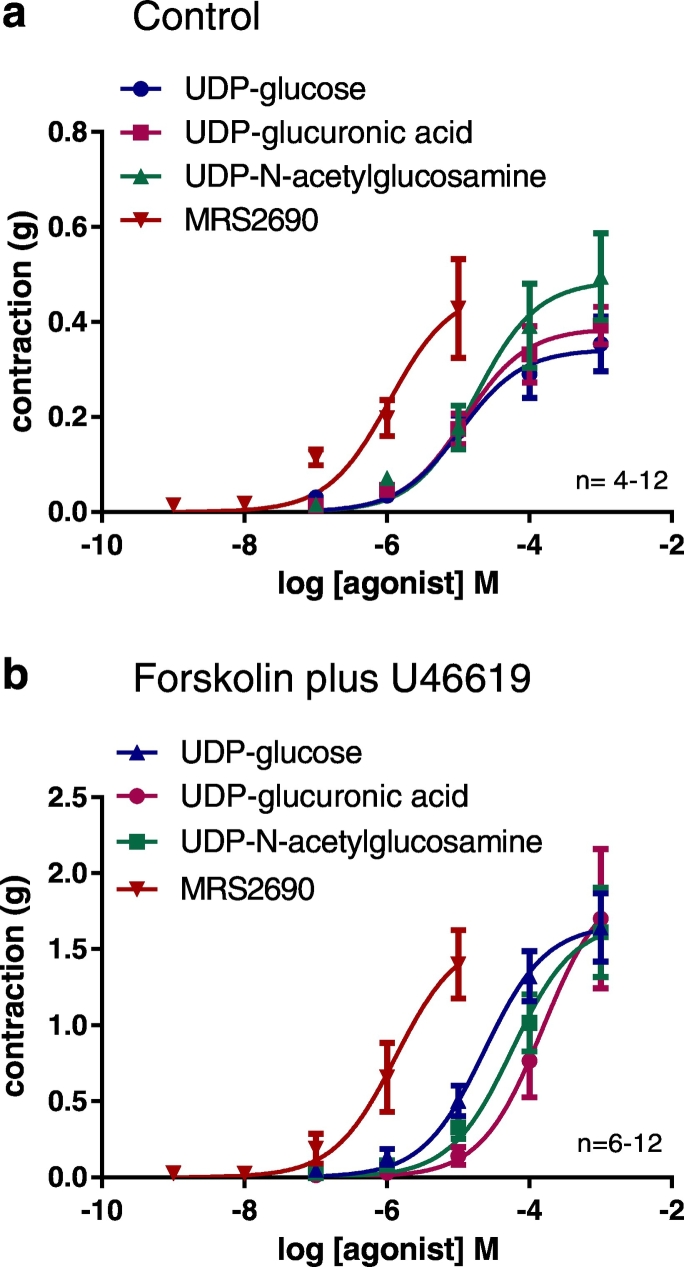
UDP-sugars mediate contraction of porcine coronary arteries and the responses are augmented by forskolin. Cumulative concentration-response curves to P2Y_14_ receptor agonists in the porcine coronary artery. UDP-glucose, UDP-glucuronic acid, UDP-*N*-acetylglucosamine and the P2Y_14_ receptor agonist MRS2690 were investigated in isolated porcine coronary arteries under control conditions (a) and in arteries to which forskolin (0.1 μM) and U46619 had been added to elevate cAMP and maintain tone, respectively (b). Note the different y axes scales in panels a and b. There was a significant difference in the agonist logEC_50_ values in the presence of forskolin plus U46619 (*P* < 0.001, extra sum of squares F test) (see also Table 1). Data are mean ± S.E.M.

UDP-glucuronic acid and UDP-*N*-acetylglucosamine (0.1 μM–1 mM) also produced concentration-dependent contractions of the porcine coronary arteries (Fig. 3; Table 1). All four of the P2Y_14_ receptor agonists (UDP-glucose, UDP-glucuronic acid, UDP-*N*-acetylglucosamine and MRS2690) produced greater contractions in tissues exposed to forskolin (and U46619) (Fig. 3b) than in control tissues (Fig. 3a), although analysis of the R_max_ values showed that this difference was only statistically significant for UDP-glucose and UDP-glucuronic acid (ANOVA, Table 1). An increase in contractility under these experimental conditions is consistent with an involvement of G_i/o_ protein coupled receptors and inhibition of cAMP. There was a significant difference between the logEC_50_ values of the agonists (*P* < 0.001), but their R_max_ values were similar (*P* > 0.05) (extra sum of squares F-test). Analysis of the logEC_50_ values (ANOVA with Tukey's post test) showed that MRS2690 was significantly more potent than the other UDP-sugars (Table 1). UMP (0.1 μM − 1 mM) did not cause contraction of the porcine coronary arteries (tested in the presence of forskolin and U46619; data not shown).

**Fig. 4:**
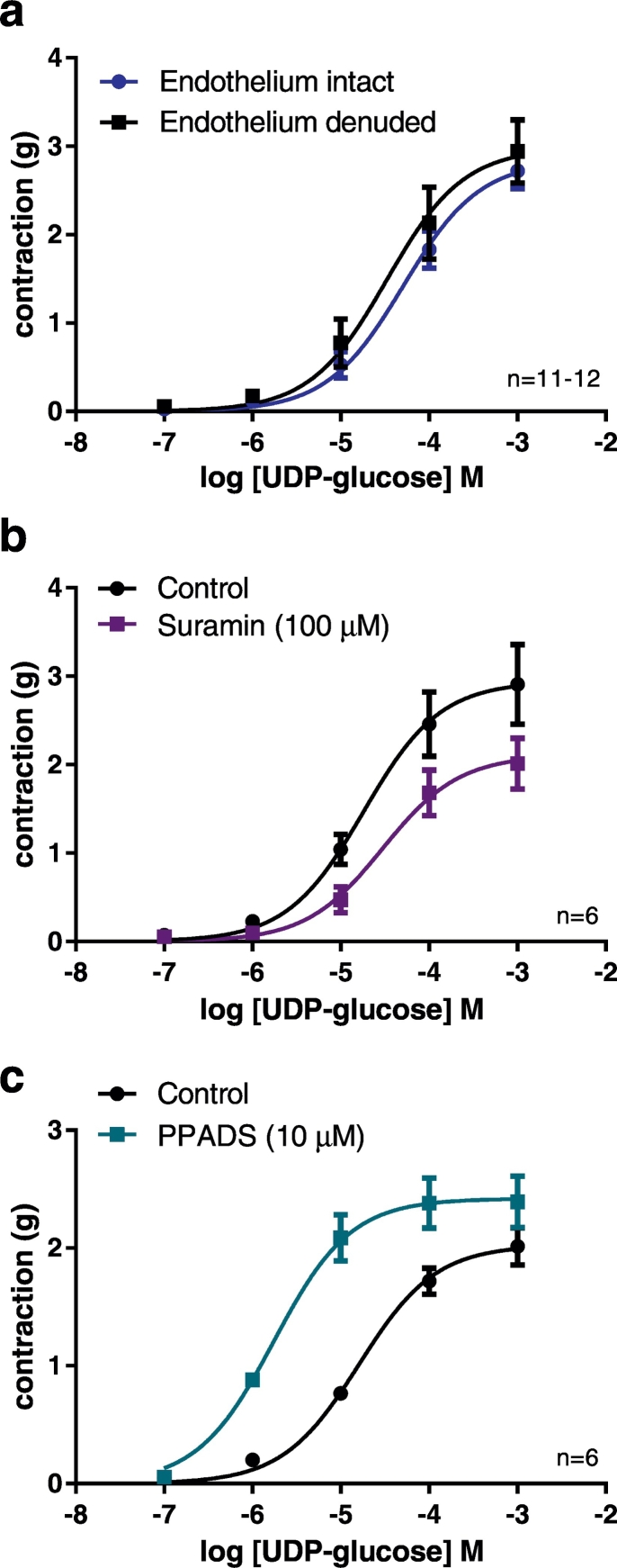
Contraction to UDP-glucose in porcine coronary arteries is endothelium-independent and attenuated by suramin but augmented by PPADS. Concentration-response curves to UDP-glucose in endothelium-intact and endothelium-denuded arteries in the presence of forskolin plus U46619 (a). Effect of the P2 receptor antagonists suramin (b) and PPADS (c) on contractile responses to UDP-glucose in porcine coronary arteries in the presence of forskolin plus U46619. UDP-glucose R_max_ values were significantly different in the presence and absence of suramin (P < 0.01) and both R_max_ and logEC_50_ values were significantly different in the presence and absence of PPADS (P < 0.01 and P < 0.001) (extra sum of squares F test). Data are mean ± S.E.M.

Removal of the endothelium had no significant effect on the contractile response to UDP-glucose in the porcine coronary artery, showing that this was mediated by direct actions on the smooth muscle (Fig. 4a); R_max_ values were 2.96 ± 0.22 g (*n* = 12) and 3.12 ± 0.36 g (*n* = 11) in endothelium-intact and -denuded arteries, respectively, and logEC_50_ values were −4.25 ± 0.13 (n = 12) and −4.34 ± 0.14 (n = 11), respectively. Substance P (10 nM) was used to confirm the presence of the endothelium in the porcine coronary arteries: in endothelium-intact arteries relaxation was 41 ± 6.4% (n = 12); in endothelium-denuded arteries substance P did not cause a relaxation (n = 11).

### 3.2 Effects of suramin and PPADS, P2 receptor antagonists, on contractile responses to UDP-glucose in coronary arteries

The P2 receptor antagonist suramin (100 μM) reduced the maximal contractile response to UDP-glucose (obtained in the presence of forskolin plus U46619) from 2.94 ± 0.45 g (*n* = 7) to 2.12 ± 0.29 g (*n* = 8) (*P* < 0.01, extra sum of squares F test) (Fig. 4b). The logEC_50_ values were not significantly different at −4.74 ± 0.04 (n = 7) and −4.50 ± 0.07 (n = 8), in the absence and presence of suramin respectively. These data are consistent with an involvement of P2 receptors as mediators of the contractile response to UDP-glucose, but do not indicate which P2 receptor subtype(s) are involved. In contrast, PPADS (10 μM) augmented the contractile response to UDP-glucose (obtained in the presence of forskolin plus U46619) (Fig. 4c); the R_max_ was significantly greater in the presence of PPADS at 2.42 ± 0.23 g (*n* = 6) than in the control at 2.03 ± 0.16 g (n = 6) (*P* < 0.01, extra sum of squares F test) and the sensitivity of the response to UDP-glucose was also increased in the presence of PPADS, logEC_50_ = −5.77 ± 0.05 (n = 6) compared to the control, logEC_50_ = −4.81 ± 0.08 (n = 6) (*P* < 0.001, extra sum of squares F test).

### 3.3 PPTN, a selective P2Y_14_ receptor antagonist, blocks responses to MRS2690, a selective P2Y_14_ receptor agonist, in coronary arteries

**Fig. 5:**
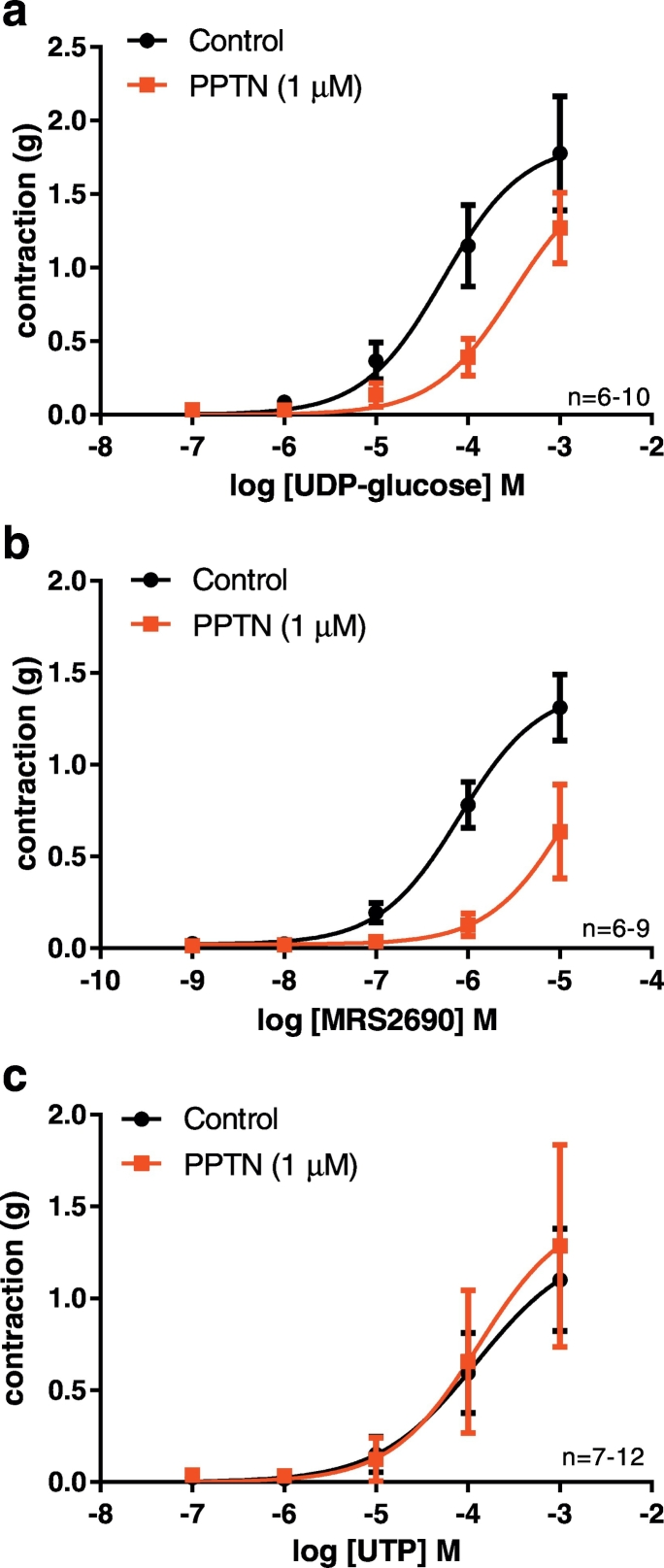
Contractile responses to UDP-glucose and the selective P2Y_14_ receptor agonist MRS2690 are attenuated by the selective P2Y_14_ receptor antagonist PPTN. The effect of PPTN (1 μM) on contractile responses to UDP-glucose (a), MRS2690 (b), and UTP (c) in porcine coronary arteries in the presence of forskolin (0.1 μM) and U46619. Analysis of the curves showed that these were significantly different in the absence and presence of PPTN for UDP-glucose (P < 0.01) and MRS2690 (P < 0.001) (extra sum of squares F test). In contrast, there was no significant difference in the responses to UTP. Data are mean ± S.E.M.

In order to investigate the possible involvement of the P2Y_14_ receptor in the porcine coronary artery, PPTN was used to examine contractile responses to the P2Y_14_ receptor agonists UDP-glucose and MRS2690. PPTN at 1 μM acts selectively to inhibit the P2Y_14_ receptor with no effect at the other P2Y receptor subtypes [6]. In the presence of PPTN (1 μM), the contractile responses to UDP-glucose were attenuated and there was a significant difference between the response curves (P < 0.01, extra sum of squares F-test) (Fig. 5a). The contractions to MRS290 were also attenuated by PPTN with a significant difference between the response curves (P < 0.001, extra sum of squares F-test) (Fig. 5b).

UTP is an agonist at P2Y_2_ and P2Y_4_ receptors, and elicits contraction of porcine coronary arteries through activation of these receptors [41]. UTP does not have any effect at the P2Y_14_ receptor [9]. To determine the selectivity of action of PPTN versus other P2Y receptors in the PCA, namely P2Y_2_ and P2Y_4_ receptors, the effect of PPTN on contractile responses to UTP was investigated. PPTN had no significant effect on the contractile responses to UTP (Fig. 5c).

### 3.4 MRS2578, a selective P2Y_6_ receptor antagonist, does not block responses to MRS2690 and UDP-glucose in coronary arteries

**Fig. 6:**
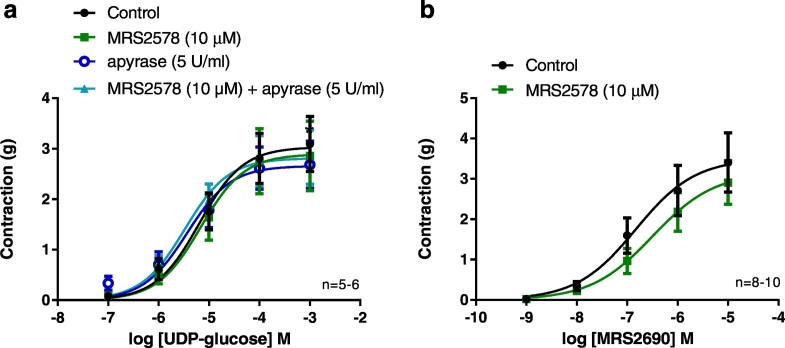
Contractile responses to UDP-glucose and MRS2690 are unaffected by the selective P2Y_6_ receptor antagonist MRS2578. Effect of MRS2578, on concentration-response curves to (a) UDP-glucose and (b) MRS2690 in porcine coronary arteries. UDP-glucose and MRS260 caused concentration-dependent contractions in the presence of forskolin and U46619. Contractions to UDP-glucose were unaffected by MRS2578 or by prior incubation of the UDP-glucose solution and tissues with apyrase (5 U/ml, > 30 min) (a). Contractions to MRS2690 were also unaffected by MRS2578. Data are mean ± S.E.M.

P2Y_6_ receptors are present in the porcine coronary artery and, upon activation by their endogenous ligand UDP, elicit vasoconstriction [41]. We investigated whether UDP-glucose and MRS2690 cause a P2Y_6_ receptor mediated contractile response in porcine coronary arteries. In addition, in order to determine whether the contractions to UDP-glucose involved UDP, either present as a contaminant or following release from the tissue, the UDP-glucose solution was treated with the nucleotidase apyrase (5 U/ml, 30 min) and apyrase was also added to the organ baths (5 U/ml, prior to addition of forskolin and U46619). We found that neither MRS2578 (10 μM), a selective P2Y_6_ receptor antagonist, nor apyrase, had any significant effect on contractile responses induced by UDP-glucose, in coronary arteries in the presence of forskolin and U46619 (Fig. 6a). UDP-glucose R_max_ values were: 2.93 ± 0.49 g (*n* = 7), 2.92 ± 0.70 g (*n* = 6), 2.68 ± 0.46 g (n = 6) and 2.81 ± 0.54 g (*n* = 5), in controls and in the presence of MRS2578, apyrase and MRS2578 plus apyrase, respectively; the corresponding logEC_50_ values were −5.26 ± 0.11, −5.45 ± 0.28, −5.48 ± 0.16, and −5.58 ± 0.16. MRS2578 also had no significant effect on contractions mediated by MRS2690 in coronary arteries in the presence of forskolin and U46619, in line with the selectivity of this compound for P2Y_14_ receptors; R_max_ values were 3.38 ± 0.66 g (*n* = 8) in controls and 2.71 ± 0.54 g (*n* = 10) in the presence of MRS2578 and the corresponding logEC_50_ values were −6.76 ± 0.22 and −6.70 ± 0.13 (Fig. 6b).

### 3.5 P2Y_14_ receptor agonists decrease VASP-phosphorylation in coronary arteries

**Fig. 7:**
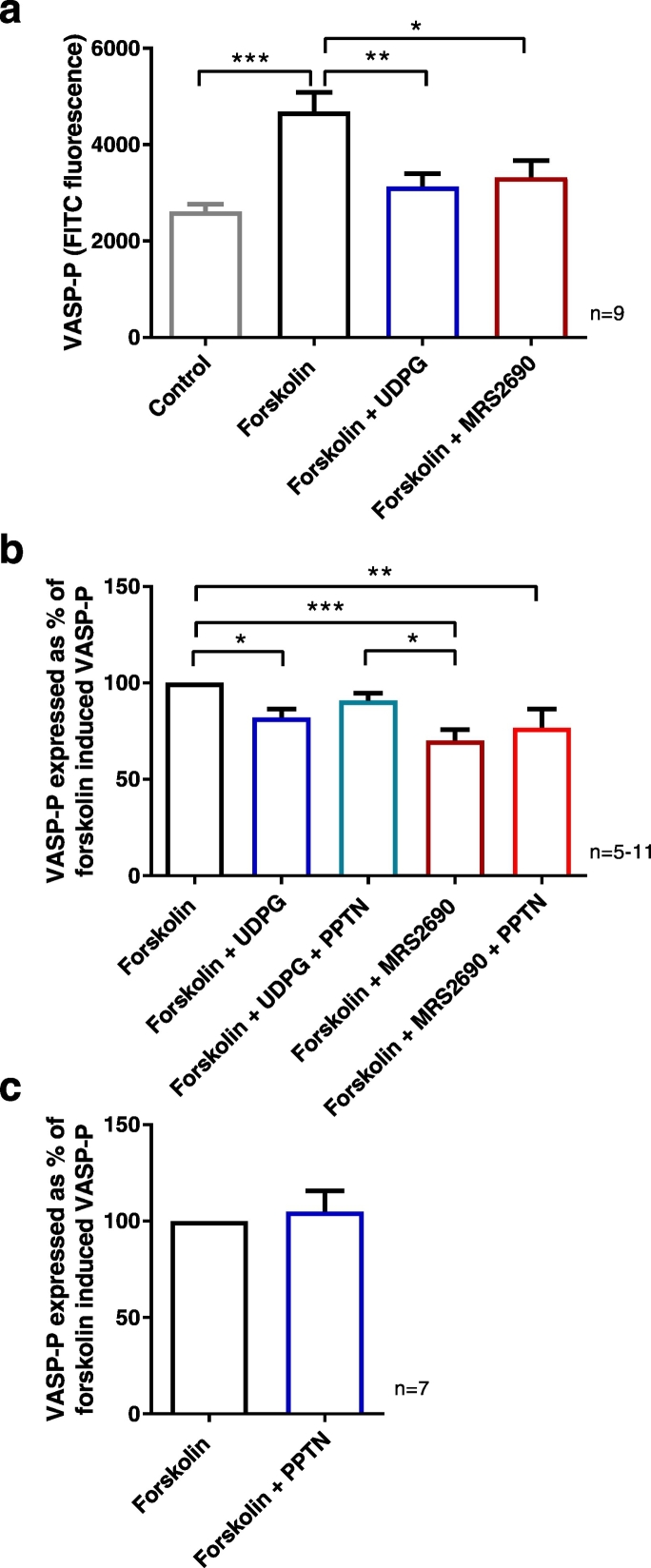
UDP-glucose-induced VASP-phosphorylation is blocked by the P2Y_14_ receptor antagonist PPTN. Levels of VASP phosphorylation (VASP-P) in porcine coronary arteries at basal tone, in the absence (Control) and presence of forskolin and the effect of UDP-glucose (UDPG), MRS2690 (P2Y_14_ receptor agonist) and PPTN (P2Y_14_ receptor antagonist). (a) Levels of VASP-P in control conditions and in the presence of forskolin, forskolin + UDP-glucose (100 μM), and forskolin + MRS2690 (10 μM). (b) Effect of UDP-glucose (1 mM) and MRS2690 (10 μM) on forskolin-induced VASP phosphorylation in porcine coronary arteries in the absence and presence of PPTN (1 μM). (c) Effect of PPTN (1 μM) on forskolin-induced VASP-P. VASP-phosphorylation was measured using flow cytometry. *P < 0.05, **P < 0.01; ***P < 0.001, ANOVA with Tukey's post test. PPTN alone had no effect on VASP-P (Student's *t*-test). Data are mean ± S.E.M.

Increases in intracellular cAMP levels lead to the phosphorylation of vasodilator-stimulated phosphoprotein (VASP) via cAMP-dependent protein kinase A (PKA) and other kinases. Thus VASP-phosphorylation (VASP-P) can be used to measure levels of cAMP. In the present study, forskolin was used to increase cAMP levels in porcine coronary arteries. Forskolin alone (0.1 μM), an activator of adenylate cyclase, significantly increased VASP-P levels (*P* < 0.001, ANOVA with Tukey's post test) (Fig. 7a). UDP-glucose (100 μM) and MRS2690 (10 μM) significantly decreased the forskolin-elevated levels of VASP-P, to levels that were similar to those measured in control tissues (*P* < 0.05, ANOVA with Tukey's post test) (Fig. 6a). PPTN (1 μM) was able to partially restore levels of VASP-P that were decreased by UDP-glucose and MRS2690 in porcine coronary arteries (Fig. 7b). PPTN alone (1 μM) had no significant effect on VASP-P levels in forskolin exposed porcine coronary arteries (Fig. 7c). Together these results suggest that P2Y_14_ receptor activation couples to the inhibition of adenylate cyclase activity, hence reducing levels of cAMP and VASP-P in porcine coronary arteries in vitro.

### 3.6 Effect of platelets in porcine coronary arteries and the effect of PPTN

**Fig. 8:**
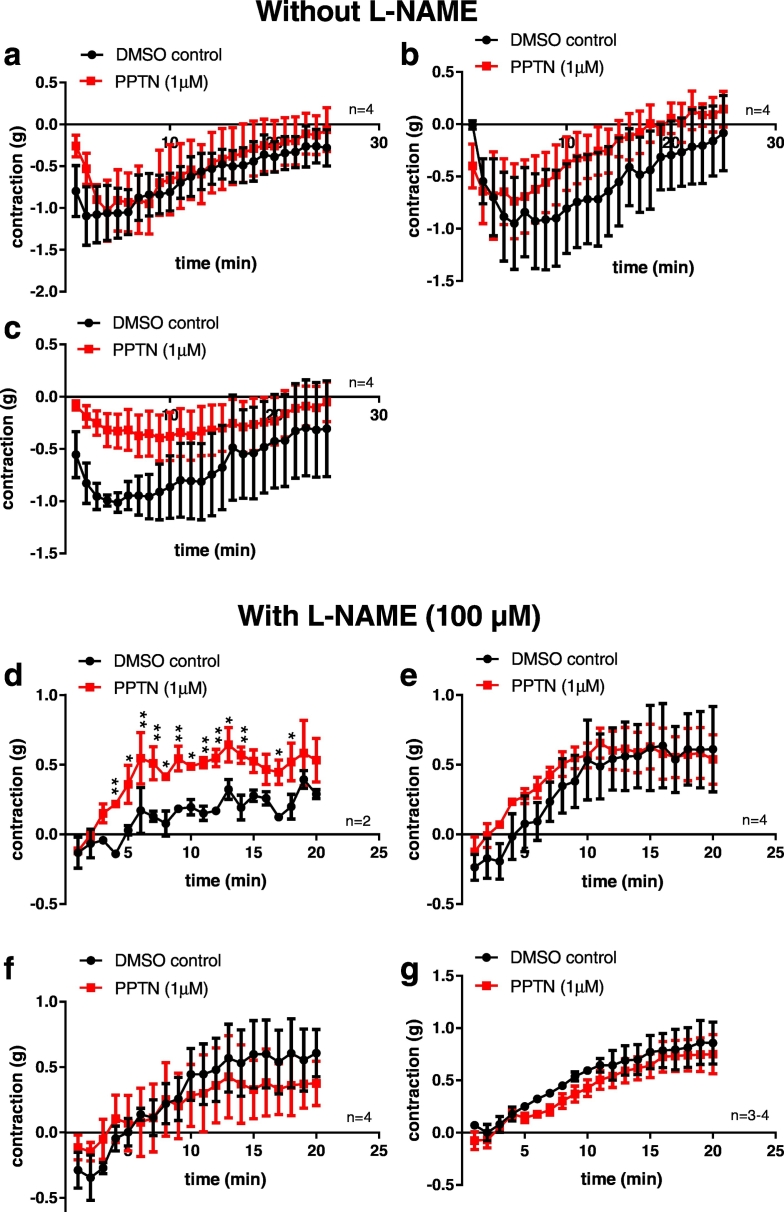
Effect of platelets and the P2Y_14_ receptor antagonist PPTN, on porcine coronary artery contractility. Platelets isolated from 7 healthy adult human donors were added to U46619-preconstricted porcine coronary arteries in the absence (a–c) and presence (d–g) of L-NAME (100 μM), a nitric oxide synthase inhibitor, in the absence and presence of PPTN (1 μM) (P2Y_14_ receptor antagonist). In the absence of L-NAME the platelets elicited vasorelaxation. In the presence of L-NAME platelets elicited vasoconstriction. n indicates the number of different pigs from which coronary artery segments were obtained. *P < 0.05; **P < 0.01 for comparison of responses in the absence and presence of PPTN (two way ANOVA). Data are mean ± S.E.M.

Isolated platelets (24,700–30,000 cells/μl) added to coronary artery segments that had been preconstricted with U46619 caused a significant relaxation (*P* < 0.01, two-way ANOVA) which reversed over time (Fig. 8a–c). PPTN (1 μM) did not significantly change the mean relaxation response induced by the platelets from 3 donors (Fig. 8a–c). L-NAME (100 μM), a nitric oxide synthase inhibitor, was added to vessels to remove the platelet-induced relaxations, and in its presence platelets caused predominantly contraction (*P* < 0.001, two-way ANOVA) (Fig. 8d-g). PPTN had no significant effect on the mean platelet-induced contractions in the presence of L-NAME in 3 donors (Fig. 8e–g), although for one donor the platelet-induced contractile response was augmented (two-way ANOVA) (Fig. 8d).

## 4 Discussion

We have provided the first evidence for the functional expression of the P2Y_14_ purine receptor in the porcine coronary vasculature. Vasoconstriction was produced by UDP-glucose, UDP-glucuronic acid, UDP-*N*-acetylglucosamine and MRS2690, all of which are known P2Y_14_ receptor agonists [4], [9], and MRS2690 was more potent than the UDP-sugars, consistent with previous characterization of MRS2690 as a potent and selective P2Y_14_ receptor agonist [31]. Responses to UDP-glucose and MRS2690 were selectively antagonized by PPTN, a competitive antagonist at the P2Y_14_ receptor [6], [43], but were unaffected by the P2Y_6_ receptor antagonist MRS2578.

Forskolin is routinely added to cultured and dissociated cells to elevate cAMP levels in order to reveal the function of G_i/o_ protein-coupled receptors, and although less commonly used in intact tissue it has been used to uncover vasocontractile α_2_-adrenoceptors in the porcine ear artery [42] and P2Y_14_ receptors in the porcine pancreatic artery [5]. Vasocontractile responses to the endogenous and synthetic P2Y_14_ receptor agonists were increased in vessels in the presence of forskolin, but not when the forskolin was substituted with sodium nitroprusside (increases cGMP). Moreover, the combination of U46619 and forskolin had no effect on contractions to histamine and acetylcholine, which in porcine coronary arteries are mediated through activation of H_1_ histamine receptors [22] and M_3_ muscarinic receptors [13] respectively; both receptors are coupled to G_q/11_ proteins and therefore do not inhibit adenylyl cyclase activity, suggesting that forskolin treatment was not augmenting responses to the vasoconstrictors non-selectively. These observations are consistent with the involvement of G_i/o_ protein-coupled P2Y_14_ receptors causing inhibition of adenylyl cyclase activity and a reduction in cAMP. Pertussis toxin treatment (which blocks G_i/o_ protein-coupling) inhibits UDP-glucose binding to P2Y_14_ receptors [4], [9], but we were not able to use pertussis toxin successfully in our studies, possibly because of difficulties with it permeating the multilayered tissue. Physiologically, elevations in cAMP levels might occur following activation of G_s_ protein-coupled β-adrenoceptors and A_2A_ and A_2B_ adenosine receptors and concomitant UDP-sugar activation of P2Y_14_ receptors would be expected to limit vasodilatation, and other functions, mediated by these receptors.

The selective P2Y_14_ receptor agonist MRS2690 was more potent than UDP-glucose, UDP-glucuronic acid and UDP-*N*-acetylglucosamine, consistent with the literature [16], [28]. Contractions to MRS2690 were evident at concentrations equal to and below 10 μM, and at 10 μM MRS2690 is inactive at P2Y_2_ receptors [30]. PPTN, which inhibits P2Y_14_ receptors but not other P2Y receptors [6], [43], blocked contractile responses of the porcine coronary artery to MRS2690 and the responses were virtually abolished at 1 μM MRS2690. Contractile responses to UTP (agonist at P2Y_2_ and P2Y_4_ receptors) were unaffected by PPTN at a concentration that inhibited responses to MRS2690. Moreover, the contractile response to MRS2690 was unaffected by the P2Y_6_ receptor antagonist MRS2578 [37]. These results indicate that P2Y_14_ receptors are functionally expressed in porcine coronary arteries, along with P2Y_2,_ P2Y_4_ and P2Y_6_ receptors, for which the principal endogenous ligands are UTP and ATP (P2Y_2_), UTP (P2Y_4_) and UDP (P2Y_6_) [41].

With regard to the endogenous ligands, contractile responses to UDP-glucose were mimicked by UDP-glucuronic acid and UDP-*N*-acetylglucosamine, consistent with an involvement of P2Y_14_ receptors. This is unlike UDP-glucose responses in N9 microglia which were not mimicked by UDP-galactose and thus were suggested to be independent of P2Y_14_ receptors [7]. Moreover, responses to UDP-glucose were blocked by PPTN, indicating actions at P2Y_14_ receptors, and consistent with a role for UDP sugars as extracellular signalling molecules. Contractile responses to UDP-glucose were also attenuated by the non-selective P2 receptor antagonist suramin. The non-selective P2 receptor antagonist PPADS augmented contractile responses to UDP-glucose, as reported by us previously in porcine pancreatic arteries [5]; the mechanism remains to be determined but does not appear to involve the ectonucleotidase inhibitory activity of PPADS [10], [20] and potentiation of responses to contaminant nucleotides, since our experiments with apyrase indicate that nucleotides are not present as major contaminants of UDP-glucose, as reported by others [47]. There is evidence for interactions between cAMP and Ca^2 +^ signalling pathways such that an increase in intracellular Ca^2 +^ inhibits cAMP accumulation [11]. There is also evidence that PPADS antagonises IP_3_-induced intracellular Ca^2 +^ mobilization [50] and that it elevates basal cAMP levels [40]. Thus, it is possible that a PPADS-induced elevation of cAMP levels (further to the elevation caused by forskolin), possibly involving a reduction in intracellular Ca^2 +^, leads to its facilitation of contractile responses to UDP-glucose.

Actions at multiple P2Y receptor targets is common among purine/pyrimidine nucleotides [8] but responses to UDP-glucose were not inhibited by MRS2578 indicating a lack of involvement of vasocontractile P2Y_6_ receptors [41]. Another possible target is the P2Y_2_ receptor, at which UDP-glucose is known to act, albeit weakly [30], and which is present in the porcine coronary artery [41]. In coronary and basilar arteries from P2Y_2_ knockout mice the response to UDP-glucose was slightly, but not significantly, smaller than that in arteries from wild type mice [21]. UMP and uridine are known not to contribute to P2Y_14_ receptor activation [9], and we found that UMP did not contract porcine coronary arteries (unpublished observations).

Since endothelium removal had no effect on contractions to UDP-glucose, the P2Y_14_ receptors mediating this response are likely to be located on the coronary artery smooth muscle. We have shown that the P2Y_14_ receptor is located on both the smooth muscle and endothelium of porcine pancreatic arteries [5], reinforcing that P2 receptor expression can vary with blood vessel as well as species [8]. Expression of mRNA encoding P2Y_14_ was shown in rat and mouse aortic smooth muscle [19], [29]. Meister et al. [38] also reported P2Y_14_ expression in blood vessels, but mainly in veins. In immunoblotting experiments for P2Y_14_ receptor protein we showed a band at 41 kDa, which is close to the predicted molecular weight of 38 kDa for the P2Y_14_ receptor, and an additional, stronger band was seen at 61 kDa (Supplemental data). Similar sized bands (34 kDa and 56 kDa) were reported for P2Y_14_ in C6 glioma cells, and evidence was provided of a contribution of glycosylation to the higher molecular weight band [33]. However, the specificity of commercially available antibodies is a concern (see [18]) and validation of the P2Y_14_ receptor antibody is needed to understand whether these bands are the P2Y_14_ receptor.

In parallel studies, VASP-P was measured as an indicator of cAMP accumulation in the porcine coronary artery. Forskolin significantly enhanced VASP-P, and UDP-glucose and MRS2690 inhibited the forskolin-induced increase in VASP-P. Moreover PPTN was able to partially reverse the UDP-glucose and MRS2690 mediated inhibition of forskolin-induced VASP-P, consistent with an involvement of P2Y_14_ receptors mediating a reduction in cAMP levels. PPTN was similarly shown to antagonise UDP-glucose-mediated inhibition of adenylyl cyclase activity in C6 glioma cells expressing P2Y_14_ receptors [6]. UDP-glucose also inhibited forskolin-stimulated cAMP accumulation in T-lymphocytes, neutrophils and human astrocytoma cells [44], [45], [46]. Overall, the data suggest that as in other tissues, the P2Y_14_ receptor in the porcine coronary artery is G_i/o_ protein-coupled.

The present findings are important because there is evidence that UDP-glucose may be a more stable extracellular signalling molecule than ATP and UTP, both of which have been shown to be released from heart during coronary hypoxia/ischaemia [14], [15], [24]. Release of UDP-glucose has been shown from several cell types (including human astrocytoma cells, Calu-3 airway epithelial cells, COS-7, CHO-K1 and C6 glioma cells) and, significantly, unlike ATP levels, the stimulated levels of UDP-glucose remained elevated for up to 3 h [36]. Similarly, there was negligible metabolism of extracellular UDP-glucose in neutrophil suspensions at 1 h, at which time ATP had been completely hydrolysed by neutrophil NTPDase1 (nucleotide triphosphate diphosphohydrolase-1), which hydrolyses extracellular nucleotides but not UDP-sugars [47]. This has important implications for P2Y_14_ receptor signalling during cardiac hypoxia, ischaemia, inflammation, or cell damage, during which UDP-glucose may be released within the coronary vasculature. UDP-glucose levels of ~ 90 μM were determined in heart extracts [34] and are in line with the effective concentrations of UDP-glucose producing vasocontraction of the coronary arteries in the present study. Such levels might be found following exocytotic release of UDP-glucose or upon cell damage in the heart or following hypoxia, ischaemia or inflammation. Other potential sources of UDP-sugars in the cardiovascular system include platelets, leukocytes and endothelial cells. It follows that antagonists at P2Y_14_ receptors may have therapeutic potential in ischaemic heart disease.

UDP-sugars including UDP-glucose, UDP-galactose, UDP-glucuronic acid and UDP-*N*-acetylglucosamine have been measured in platelets [51] and we were interested to see if UDP-sugars are released from activated platelets to mediate contraction of coronary arteries. In the porcine coronary arteries the addition of platelets from the human donors produced a relaxation, which was unaffected by PPTN. In canine coronary arteries, aggregating platelets cause an endothelium-dependent relaxation which is largely due to the actions of ADP and ATP released from the platelets and this relaxation was reduced in vessels treated with apyrase [25], [26]. In endothelium denuded canine coronary arteries, platelets elicit vasoconstriction that does not occur in endothelium intact vessels [26], [48]. Thus, we used an inhibitor of nitric oxide synthase, L-NAME, to uncover the contractile response to the platelets. This is the first study to use L-NAME to block platelet-induced relaxations in vascular tissue and thus to show a specific involvement of NO. The porcine coronary arteries contracted when platelets were added in the presence of L-NAME, likely due to actions of contractile mediators including 5-HT and thromboxane A_2_ released from the activated platelets [26], but PPTN had no significant effect on this response in 3 of the donors and augmented the response in one, suggesting that P2Y_14_ receptors do not contribute significantly under the conditions of the present study. The effects of the UDP-sugars may be small in comparison to those of the other components released from platelets. High levels of P2Y_14_ receptor mRNA have been detected in megakaryocytic cells and platelets [39]. Although P2Y_14_ receptors have been characterised in platelets using immunoblotting analysis, UDP-glucose and MRS2690 did not have any effect on a number of human platelet functions [12].

In summary, UDP-glucose mediates contraction of the porcine coronary artery via P2Y_14_ receptors. The P2Y_14_ receptor is coupled negatively to adenylyl cyclase since UDP-glucose and the selective P2Y_14_ receptor agonist MRS2690 decreased cAMP accumulation as determined by lower levels of VASP-P; these decreased levels of VASP-P could partially be restored by PPTN, a selective P2Y_14_ receptor antagonist. P2Y_14_ receptors may be involved in the control of coronary arterial blood flow and may, therefore, be possible targets for the treatment of ischaemic heart disease.

## Funding

This work was supported by the British Heart Foundation [Grant number FS/12/7/29359].

## Conflict of interest

None declared.

## Appendix A Supplementary data

Immunoblotting with P2Y14 antibody Image 1
