# Supplementary material for: UDP-sugars activate P2Y14 receptors to mediate vasoconstriction of the porcine coronary artery
Source: Vascul Pharmacol. 2018 Apr;103-105:36–46. doi: 10.1016/j.vph.2017.12.063 (PMC5906693; doi:10.1016/j.vph.2017.12.063)
Supplement: Supplementary file 1 — Immunoblotting with P2Y14 antibody [file mmc1.pdf]

Supplemental data

Immunoblotting

Porcine coronary arteries were dissected as described above. Control tissues were brains and hearts obtained from male Wistar rats, which were put into lysis buffer (containing protease inhibitor enzyme) and frozen at -20 ^{0}C. Tissues were homogenized and the supernatant was separated by centrifugation (3000 speed, 825 RCF, for 5 min, 4 ^{0}C). A Lowry protein assay was carried out to normalize all samples to 1 mg/ml. For the western blotting procedure samples were heated to 95 ^{0}C for 5 min, vortexed and centrifuged (13,000 rpm, 1 min). Separation of proteins was performed using -20% BioRad precast polyacrylamide SDS/PAGE gels. Proteins were transferred onto nitrocellulose membranes. Membranes were blocked for 1 hour in 5% milk in TBST (0.1% tween). Blots were incubated overnight at 4^{0}C with either anti-$P2Y_{14}$ antibody or anti-GPR17 antibody. The following day, blots were incubated with secondary antibodies for anti-$P2Y_{14}$ (IRDye 800CW Goat anti-Rabbit IgG (H + L), 0.5 mg). Immunocomplexes were detected using an Odyssey scanner with image studio V3 software. Anti-$P2Y_{14}$ antibody (intracellular epitope, LS-C120603) was obtained from LifeSpan BioSciences Inc; the immunogenic sequence shows 81% identity with pig P2RY14 (F1SJN3).

Immunoblotting using $P2Y_{14}$ receptor antibody (Supplemental Figure 1a) showed the presence of two bands sized around 61 kDa and 41 kDa in coronary arteries from 3 different pigs (PCA; lanes 1, 2 and 3), pig heart (PH) (lane 4) and rat heart (RH) (lane 5; used as positive control tissue). $P2Y_{14}$ receptor mRNA has been identified in the rat heart (Musa et al., 2009) and thus this was used as a positive control tissue.

GPR17 is a non-P2Y receptor activated by UDP-glucose and cysteinyl leukotrienes (Daniele et al., 2011) and has been detected in human heart, brain and kidney (Benned-Jensen and Rosenkilde, 2010). Immunoblotting using GPR17 receptor antibody showed a band of approximately 44 kDa in rat brain, consistent with the supplier’s guidelines (but slightly higher than their reported molecular weight for GPR17 of 41 kDa) and a second band at 38 kDa (Supplemental Figure 1b). No GPR17 protein was detected in the porcine coronary artery (PCA; lanes 1, 2); rat brain (RB) was used as a positive control (lane 3). Rat heart (RH; lane 4).

Benned-Jensen T, Rosenkilde MM. Distinct expression and ligand-binding profiles of two consitiutively active GPR17 splice variants. *Br J Pharmacol* 2010;**159**:1092-1105.

Daniele S, Trincavelli ML, Gabelloni P, Lecca D, Rosa P, Abbracchio MP, Martini C. Agonist-induced desensitization/resensitization of human G protein-coupled receptor 17: a functional cross-talk between purinergic and cysteinyl-leukotriene ligands. *J Pharmacol Exp Ther* 2011;**338(2)**:559-567.

Musa H, Tellez JO, Chandler NJ, Greener ID, Maczewski M, Mackiewicz U et al. P2 purinergic receptor mRNA in rat and human sinoatrial node and other heart regions. *Naunyn Schmied Arch Pharmacol* 2009;**379(6)**:541-549.

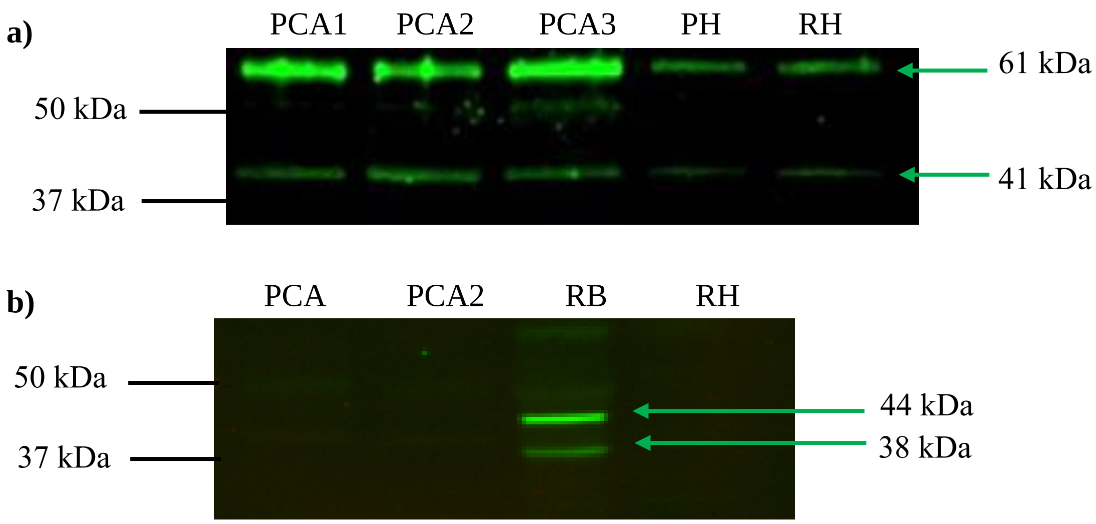

Supplemental Figure 1
